# Mechanisms of phase‐3 early afterdepolarizations and triggered activities in ventricular myocyte models

**DOI:** 10.14814/phy2.14883

**Published:** 2021-06-10

**Authors:** Zhaoyang Zhang, Zhilin Qu

**Affiliations:** ^1^ Department of Medicine David Geffen School of Medicine University of California Los Angeles CA USA; ^2^ Department of Computational Medicine David Geffen School of Medicine University of California Los Angeles CA USA

**Keywords:** afterdepolarizations, model

## Abstract

Early afterdepolarizations (EADs) are abnormal depolarizations during the repolarizing phase of the action potential, which are associated with cardiac arrhythmogenesis. EADs are classified into phase‐2 and phase‐3 EADs. Phase‐2 EADs occur during phase 2 of the action potential, with takeoff potentials typically above −40 mV. Phase‐3 EADs occur during phase 3 of the action potential, with takeoff potential between −70 and −50 mV. Since the amplitude of phase‐3 EADs can be as large as that of a regular action potential, they are also called triggered activities (TAs). This also makes phase‐3 EADs and TAs much more arrhythmogenic than phase‐2 EADs since they can propagate easily in tissue. Although phase‐2 EADs have been widely observed, phase‐3 EADs and TAs have been rarely demonstrated in isolated ventricular myocytes. Here we carry out computer simulations of three widely used ventricular action potential models to investigate the mechanisms of phase‐3 EADs and TAs. We show that when the T‐type Ca^2+^ current (I_Ca,T_) is absent (e.g., in normal ventricular myocytes), besides the requirement of increasing inward currents and reducing outward currents as for phase‐2 EADs, the occurrence of phase‐3 EADs and TAs requires a substantially large increase of the L‐type Ca^2+^ current and the slow component of the delayed rectifier K^+^ current. The presence of I_Ca,T_ (e.g., in neonatal and failing ventricular myocytes) can greatly reduce the thresholds of these two currents for phase‐3 EADs and TAs. This implies that I_Ca,T_ may play an important role in arrhythmogenesis in cardiac diseases.

## Graphical abstract

Phase‐3 early afterdepolarizations (EADs) and triggered activities (TAs) are widely observed in Purkinje fiber cells but rarely in isolated ventricular myocytes. Computer simulations using ventricular action potential models are carried out to investigate the underlying mechanisms of phase‐3 EADs and TAs. In the absence of I_Ca,T_, such as in normal ventricular myocytes, unrealistically large I_Ca,L_ and I_Ks_ conductances are needed for phase‐3 EADs and TAs to occur. However, the presence of I_Ca,T_ can substantially lower these conductance thresholds. Unlike phase‐2 EADs which are suppressed by increasing I_Ks_, increasing I_Ks_ promotes phase‐3 EADs and TAs. I_Ca,T_ is a key current for the genesis of phase‐3 EADs and TAs and may play an important role in arrhythmogenesis in cardiac diseases, such as in heart failure in which I_Ca,T_ is present.

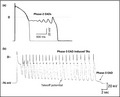

## 1 INTRODUCTION

**FIGURE 1:**
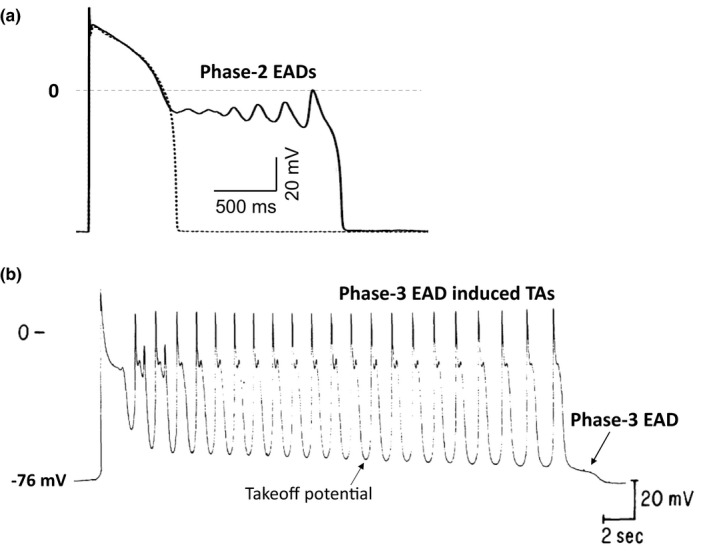
Phase‐2 and phase‐3 EADs recorded in experiments. (a). Phase‐2 EADs recorded from an isolated rabbit ventricular myocyte (Liu et al., 2012). (b). Phase‐2 and phase‐3 EADs, and phase‐3 EAD induced triggered activities recorded from an isolated canine Purkinje fiber (Damiano & Rosen, 1984)

Early afterdepolarizations (EADs), which are associated with lethal arrhythmias in cardiac diseases (Cranefield & Aronson, 1988; El‐Sherif et al., 1990; Rosen et al., 1984; Volders et al., 2000; Vos et al., 2000; Weiss et al., 2010), are abnormal depolarizations during the plateau or repolarizing phase of an action potential (AP). Early afterdepolarizations (EADs) are traditionally classified into phase‐2 EADs and phase‐3 EADs. Phase‐2 EADs are depolarizations during the plateau phase or phase 2 of the AP with takeoff potentials typically above −40 mV. Figure 1a shows an AP with normal repolarization (dashed line) and an AP with phase‐2 EADs (solid line) recorded from an isolated rabbit ventricular myocyte (Liu et al., 2012). In the one with EADs, when the voltage decays to below 0 (~−10 mV), it begins to oscillate, resulting in EADs. The takeoff potential of the EADs decreases and the amplitude increases to a maximum before repolarizing to the resting potential. The lowest takeoff potential is around −20 mV. Phase‐3 EADs are depolarizations during phase 3 of the AP with takeoff potentials typically ranging from −50 to −70 mV. Figure 1b shows an example of phase‐3 EADs recorded from an isolated Purkinje fiber (Damiano & Rosen, 1984). Traditionally, the large depolarizations are called phase‐3 EAD‐induced triggered activities (TAs). The ones with small depolarization amplitudes (even if there is no depolarization but just a slow repolarization, such as the last one in Figure 1b) are called phase‐3 EADs (Damiano & Rosen, 1984; Szabo et al., 1994, 1995). Similar to that of phase‐2 EADs, the takeoff potential also decreases with time, but the voltage of each TA reaches the same peak.

Due to the difference in their takeoff potentials and amplitudes, phase‐2 EADs and phase‐3 EADs and TAs exhibit different arrhythmogenic consequences. For example, experimental studies of Purkinje fibers have shown that phase‐2 EADs cannot conduct, but phase‐3 EAD induced TAs can conduct to form PVCs and trigger ventricular arrhythmias (El‐Sherif et al., 1990; Kupersmith & Hoff, 1985; Méndez & Delmar, 1985), indicating the importance of phase‐3 EADs and TAs in cardiac arrhythmogenesis. The mechanisms of phase‐2 EADs have been widely investigated and well understood (Qu et al., 2013), but the mechanisms of phase‐3 EADs and TAs remain to be elucidated. Therefore, understanding the mechanisms of phase‐3 EADs and TAs may provide important insights into the genesis and prevention of cardiac arrhythmias.

Phase‐2 EADs have been widely demonstrated in experiments of isolated myocytes and intact tissue. Phase‐3 EADs and TAs have been recorded in isolated Purkinje fiber experiments (Bailie et al., 1988; Damiano & Rosen, 1984; Davidenko et al., 1989; Gilmour & Moise, 1996; Roden & Hoffman, 1985; Szabo et al., 1994, 1995), promoted by hypokalemia and block of outward currents. They were also observed in ventricular tissue in some experiments (Bailie et al., 1988; Ben‐David & Zipes, 1988; El‐Sherif et al., 1990; Hwang et al., 2020), but it is unclear if they were oscillations caused by a single‐cell mechanism or by a tissue‐scale mechanism due to repolarization gradients (Huang et al., 2016; Maruyama et al., 2011). However, phase‐3 EADs and TAs have been rarely observed in isolated ventricular myocytes [low amplitude phase‐3 EADs with take‐off potential around −50 mV but not TAs were reported in isolated mice ventricular myocytes (Edwards et al., 2014; Tazmini et al., 2020)]. This was supported by a literature survey by Huang et al (Huang et al., 2018), which showed that the vast majority of the EADs recorded in experiments of isolated ventricular myocytes are phase‐2 EADs (see table 1 in Huang et al), no phase‐3 EADs and TAs were observed. Moreover, phase‐3 EADs and TAs were also not observed in computer simulations of ventricular AP models, and only phase‐2 EADs were reported [see EAD takeoff potentials in our previous study for different AP models (Huang et al., 2018), as well as EADs shown in many other computer simulation studies (Clancy & Rudy, 1999; Kurata et al., 2017, 2020; Pueyo et al., 2011; Song et al., 2015; Tanskanen et al., 2005; Varshneya et al., 2018)]. This leads to a question why phase‐3 EADs and TAs have rarely been observed in ventricular myocytes.

In this study, we carry out computer simulations of three widely used ventricular AP models to investigate the mechanisms for the genesis of phase‐3 EADs and TAs in single ventricular myocytes. We show that differing from phase‐2 EADs which are promoted by increasing inward currents (e.g., I_Ca,L_) and reducing outward currents (e.g., I_Kr_ and I_Ks_), the occurrence of phase‐3 EADs and TAs requires a reduction of I_Kr_ but a substantial increase of both I_Ca,L_ and I_Ks_. They also require a large reduction of I_K1_ and increase of I_NCX_. However, when the T‐type Ca^2+^ current, I_Ca,T_, is present (such as in neonatal or failing ventricular myocytes), the thresholds of I_Ca,L_ and I_Ks_ conductance for phase‐3 EADs and TAs can be greatly reduced. This implies that I_Ca,T_ may play an important role in arrhythmogenesis in cardiac diseases.

## 2 METHODS

Single‐cell simulations were carried out in this study. The governing differential equation for voltage (*V*) is:

(1) $$\frac{\mathrm{dV}}{\mathrm{dt}}=‐\frac{I_{\mathrm{ion}}+I_{\mathrm{stim}}}{C_{m}}$$

where *C*_m_ = 1 μF/cm^2^ and *I*_ion_ is the total ionic current density. The *I*_ion_ formulations are different for different AP models. In this study, we used the 2011 O’Hara et al (ORd) human ventricular AP model (O'Hara et al., 2011), the 2004 ten Tusscher et al (TP04) human ventricular AP model (ten Tusscher et al., 2004), and the 2004 Hund and Rudy (HRd) canine ventricular AP model (Hund & Rudy, 2004). The original codes for the AP models were downloaded from the authors’ websites and incorporated into our own C++ codes. *I*_stim_ is the stimulus current density, which is a short pulse. The durations and strengths of the stimulation pulses as well as the initial conditions were the same as in the authors’ original codes of the AP models. In all simulations, a single pacing stimulus was applied at *t *= 100 ms to elicit an AP to observe EADs.

We varied the maximum conductance of the major ionic currents in the AP models and used α to denote the fold change of a parameter from its value in the original model. For example, $\alpha \;(P_{\mathrm{Ca}})$ indicates that the maximum conductance of I_Ca,L_ is α fold of that in the original model, and thus $\alpha \;\left(P_{\mathrm{Ca}}\right)=1$ corresponds to the maximum conductance of I_Ca,L_ in the original model. To search different EAD behaviors in these AP models, we randomly draw the α values uniformly distributed in the pre‐assigned intervals for the following ionic currents: I_Ca,L_, I_Ks_, I_Kr_, I_K1_, I_to_, and I_NCX_. To ensure the occurrence of phase‐3 EADs and TAs in the model, we chose very (maybe unphysiologically) large intervals for some of the α values. For the ORd model, $\alpha \;\left(P_{\mathrm{Ca}}\right)\in \left[0,\;20\right]$ and $\alpha \;\left(G_{\mathrm{Ks}}\right)\in \left[0,\;100\right]$. For the TP04 model, $\alpha \;\left(P_{\mathrm{Ca}}\right)\in \left[0,\;20\right]$ and $\alpha \;\left(G_{\mathrm{Ks}}\right)\in \left[0,\;10\right]$. For the HRd model, $\alpha \;\left(P_{\mathrm{Ca}}\right)\in \left[0,\;50\right]$ and $\alpha \;\left(G_{\mathrm{Ks}}\right)\in \left[0,\;20\right]$. For all three models, $\alpha \;\left(G_{\mathrm{Kr}}\right)\in \left[0,\;2\right]$, $\alpha \;\left(G_{K1}\right)\in \left[0.5,\;2\right]$, $\alpha \;\left(G_{\mathrm{to}}\right)\in \left[0,\;2\right]$, and $\alpha \;\left(v_{\mathrm{NCX}}\right)\in \left[0.5,\;5\right]$.

We performed parameter sensitivity analyses for EADs using the logistic regression method proposed by Morotti and Grandi (Morotti & Grandi, 2017).

Simulations were carried out using a fixed timestep, Δ*t *= 0.01 ms. All the models were programed in CUDA C++ and simulations were performed on Nvidia Tesla K80 GPU cards (NVIDIA corporation).

## 3 RESULTS

### 3.1 Phase‐3 EADs and TAs

**FIGURE 2:**
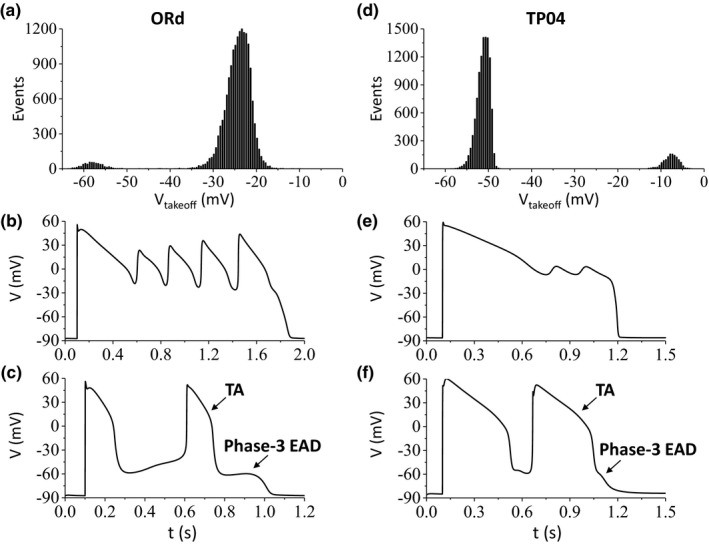
Phase‐2 EADs and phase‐3 EADs and TAs in the ORd and TP04 models. (a) Histogram of EAD takeoff potential for the ORd model obtained by randomly drawing parameters from the assigned intervals (see Methods). The total number of parameter sets resulting in phase‐2 EADs is 16,165 and that for phase‐3 EADs and TAs is 617 in this histogram. (b) An example AP exhibiting phase‐2 EADs in the ORd model. α (P_Ca_) = 11.364, α (G_Ks_) = 1.675, α (G_Kr_) = 0.858, α (G_K1_) = 0.846, α (G_to_) = 1.583, and α (v_NCX_) = 4.676. (c) An example AP exhibiting a phase‐3 EAD and TA in the ORd model. α (P_Ca_) = 17.994, α (G_Ks_) = 96.302, α (G_Kr_) = 0.003, α (G_K1_) = 0.612, α (G_to_) = 1.727, and α (v_NCX_) = 3.26. (d) Histogram of EAD takeoff potential for the TP04 model. The total number of parameter sets resulting in phase‐2 EADs is 1,295 and that for phase‐3 EADs and TAs is 10,695 in this histogram. (e) An example AP exhibiting phase‐2 EADs in the TP04 model. α (P_Ca_) = 11.023, α (G_Ks_) = 0.445, α (G_Kr_) = 0.42, α (G_K1_) = 1.926, α (G_to_) = 0.354, and α (v_NCX_) = 1.633. (f) An example AP exhibiting phase‐3 EAD and TA in the TP04 model. α (P_Ca_) = 18.808, α (G_Ks_) = 3.536, α (G_Kr_) = 0.018, α (G_K1_) = 0.611, α (G_to_) = 1.575, and α (v_NCX_) = 1.757. Events in A and B are the parameter sets giving rise to EADs. V_takeoff_ is the lowest takeoff potential for each parameter set. The takeoff potential of an EAD is defined by the voltage at which dV/dt changes from negative to positive preceding the EAD

In general, one can increase the inward currents or decrease the outward currents to promote phase‐2 EADs in many of the ventricular AP models. However, the same strategy does not work for observing phase‐3 EADs and TAs. To search for phase‐3 EADs and TAs, we randomly draw maximum conductance of the major ionic currents from assigned intervals (see Methods). For each parameter set, we detected the lowest EAD takeoff potential if EADs occurred in the AP. We plotted the histograms of EAD takeoff potentials in Figure 2a,d for the ORd model and the TP04 model, respectively. For both models, the histograms exhibit bimodal distributions, which are distinctly separated into two groups. For the ORd model (Figure 2a), the takeoff potentials of the phase‐2 EADs are between −35 and −15 mV. An example of phase‐2 EADs is shown in Figure 2b. The takeoff potentials of the phase‐3 EADs and TAs are between −65 and −50 mV. An example of phase‐3 EADs and TAs is shown in Figure 2c. For the TP04 model (Figure 2d), the takeoff potentials of the phase‐2 EADs are between −15 and 0 mV, and those of phase‐3 EADs and TAs are between −60 and −45 mV. Examples of phase‐2 EADs and phase‐3 EADs and TAs from the TP04 model are shown in Figure 2e,f, respectively. The EAD takeoff potentials from these two models agree well with those observed in experiments.

**FIGURE 3:**
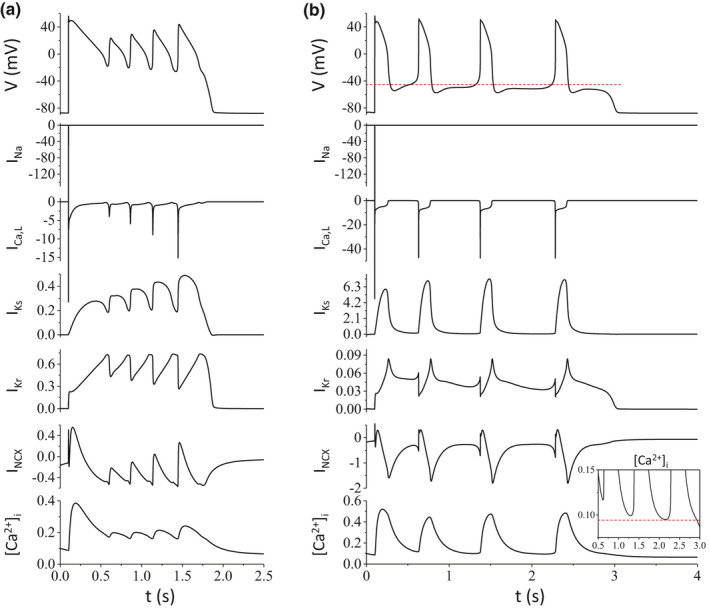
Ionic currents and Ca^2+^ transients during phase‐2 EADs and phase‐3 EADs and TAs from the ORd model. Shown are V, I_Na_, I_Ca,L_, I_Ks_, I_Kr_, I_NCX_, and [Ca^2+^]_i_ versus time. (a) Phase‐2 EADs. The parameters are the same as for Figure 2b. (b) Phase‐3 EADs. Inset in the bottom panel is an amplified view of the diastolic [Ca^2+^]_i_ from t = 0.5–3 s. The dashed horizontal lines are for references. The parameters are: α (P_Ca_) = 18.861, α (G_Ks_) = 78.447, α (G_Kr_) = 0.095, α (G_K1_) = 0.51, α (G_to_) = 1.928, and α (v_NCX_) = 4.965

To show the difference in ionic currents for the two types of EADs, we plotted the APs and the major ionic currents during an AP with phase‐2 EADs and an AP with phase‐3 EADs and TAs from the ORd model in Figure 3. For the case of phase‐2 EADs (Figure 3a), the takeoff potentials of the EADs decrease and the amplitudes of the EADs increase with time, agreeing with the general EAD features observed in experiments (e.g., Figure 1a). During the EADs, no I_Na_ is activated. The peak I_Ca,L_ increases with time and reaches maximum during the last EAD. Note that the peak I_Ca,L_ in the last EAD is much larger than that in the regular depolarization, which is a feature of the ORd model (Kurata et al., 2017; Yang et al., 2017). The peak I_Ks_ increases with time and reaches maximum in the last EAD, but the peak I_Kr_ remains unchanged. The increase of I_Ks_ is responsible for the termination of the EAD. I_NCX_ and [Ca^2+^]_i_ also oscillate during the EADs.

For the case of phase‐3 EADs and TAs (Figure 3b), the takeoff potentials decrease but only slightly (a dashed line was added on the top panel for reference), but the peak voltages remain the same. The time interval between two consecutive TAs also increases. These features agree with those observed in experiments (e.g., Figure 1b). During the phase‐3 EADs and TAs, no I_Na_ is activated. The amplitudes of other currents remain almost unchanged during the TAs (compare those in the last two TAs). However, the diastolic [Ca^2+^]_i_ becomes progressively lower during the TAs (see the inset on the bottom panel for a blowup view). The reason for this decay may be that Ca^2+^ entry is fixed (since I_Ca,L_ remains the same) but Ca^2+^ extrusion is increased due to the increase in the time interval between TAs. A lower diastolic [Ca^2+^]_i_ reduced the inward mode of NCX, which reduces I_NCX_, resulting in the lowering of the takeoff potential and eventually terminating the TAs.

### 3.2 Key ionic currents for the genesis of phase‐3 EADs and TAs

**FIGURE 4:**
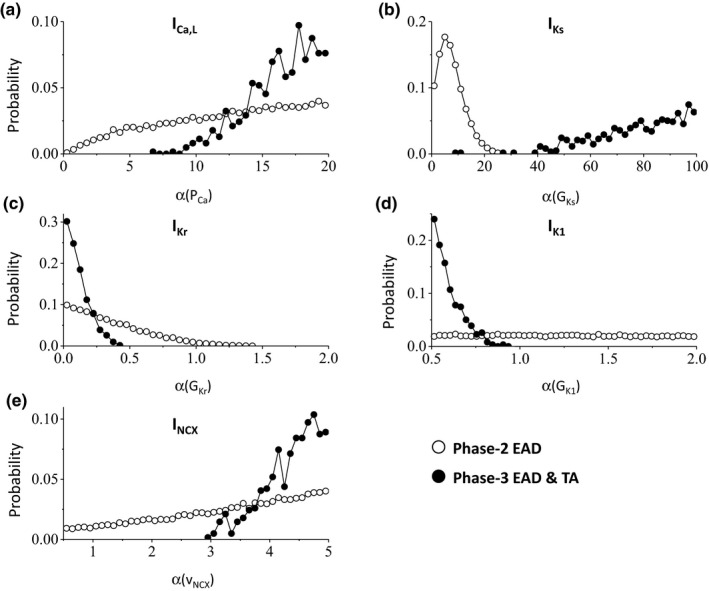
Distributions of maximum conductance for phase‐2 EADs and phase‐3 EADs and TAs in the ORd model. Data are from the same simulations as in Figure 2a. Shown are probability of events (the fraction of the parameter sets giving rise to EADs) versus α (fold of the control value of the maximum conductance) for phase‐2 EADs (open circles) and phase‐3 EADs and TAs (filled circles). (a) I_Ca,L_. (b) I_Ks_. (c) I_Kr_. (d) I_K1_. (e) I_NCX_

**FIGURE 5:**
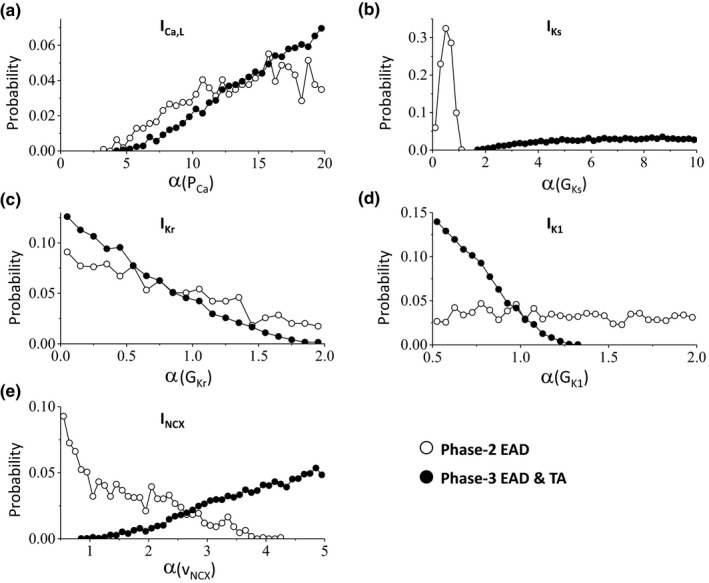
Distributions of maximum conductance for phase‐2 EADs and phase‐3 EADs and TAs in the TP04 model. Data are from the same simulations as in Figure 2d. Shown are probability of events (the fraction of the parameter sets giving rise to EADs) versus α (fold of the control value of the maximum conductance) for phase‐2 EADs (open circles) and phase‐3 EADs and TAs (filled circles). (a) I_Ca,L_. (b) I_Ks_. (c) I_Kr_. (d) I_K1_. (e) I_NCX_

Since phase‐2 EADs and phase‐3 EADs and TAs are separately grouped in the histograms shown in Figure 2, we can then separate the parameter sets for the two types of EADs. This allows us to dissect the differential contributions of the ionic currents in the genesis of phase‐2 EADs and phase‐3 EADs and TAs. Figure 4 shows the parameter (maximum conductance) distributions of phase‐2 EADs and phase‐3 EADs and TAs for the ORd model. Both types of EADs occur more frequently as the maximum I_Ca,L_ conductance increases but phase‐3 EADs and TAs requires a much higher I_Ca,L_ conductance (Figure 4a). As expected, phase‐2 EADs are promoted by reducing I_Ks_ conductance, but surprisingly phase‐3 EADs and TAs are promoted by increasing the maximum I_Ks_ conductance (Figure 4b). Both types of EADs are promoted by reducing I_Kr_ conductance but more reduction is needed for phase‐3 EADs and TAs (Figure 4c). Phase‐2 EADs are insensitive to I_K1_ but phase‐3 EADs and TAs require reduction of I_K1_ conductance (Figure 4d). Increasing NCX activity slightly increases the occurrence of phase‐2 EADs but phase‐3 EADs and TAs require a much larger I_NCX_ (Figure 4e). For the TP04 model (Figure 5), the histograms are qualitatively the same but exhibit certain quantitative difference from the ORd model. For example, phase‐3 EADs and TAs only require a slightly larger I_Ca,L_ conductance than phase‐2 EADs and increasing I_NCX_ tends to promote phase‐3 EADs and TAs but suppress phase‐2 EADs. Similarly, phase‐2 EADs are promoted by reducing I_Ks_ conductance but phase‐3 EADs and TAs are promoted by increasing I_Ks_ conductance.

**FIGURE 6:**
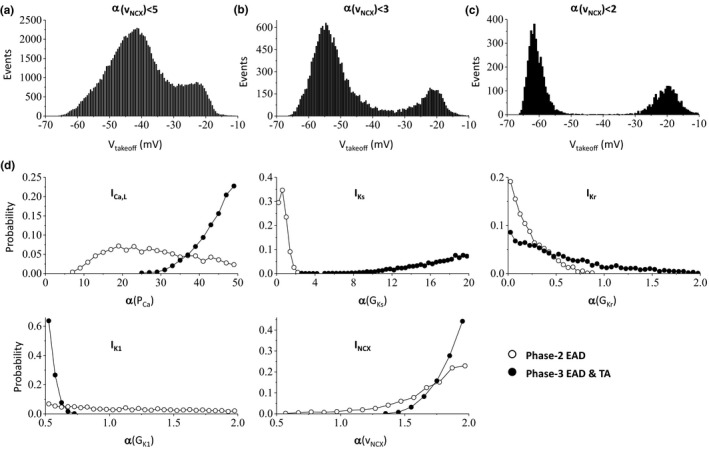
Phase‐3 EADs and TAs in the HRd model. (a) Histogram of V_takeoff_ for $\alpha \;\left(v_{\mathrm{NCX}}\right)\in \left[0.5,\;5\right]$. The total parameter sets (events) exhibiting EADs are 101,410 in this panel. (b) Histogram of V_takeoff_ for $\alpha \;\left(v_{\mathrm{NCX}}\right)\in \left[0.5,\;3\right]$. The total parameter sets (events) exhibiting EADs are 18,230 in this panel. (c) Histogram of V_takeoff_ for $\alpha \;\left(v_{\mathrm{NCX}}\right)\in \left[0.5,\;2\right]$. The total parameter sets (events) exhibiting EADs are 5,975 in this panel. (d) Probability of events (the fraction of the parameter sets giving rise to EADs) versus α (fold of the control value of the maximum conductance) for phase‐2 EADs (open circles) and phase‐3 EADs and TAs (filled circles) for I_Ca,L_, I_Ks_, I_Kr_, I_K1,_ and I_NCX_ from the same dataset in panel c

We did the same simulations for the HRd model with similar assigned intervals for the maximum conductance (see Methods). The histogram of takeoff potentials also exhibits a bimodal distribution but there is no distinct separation for the two types of EADs (Figure 6a). In this set of simulations, we used the maximum NCX activity to be 5 folds of the control value. When we allowed the maximum fold of I_NCX_ activity to be 3 folds of the control value, the bimodal distribution is more prominent (Figure 6b). When we reduced it to 2 folds, the two types of EADs are separated (Figure 6c). Using the parameter sets in Figure 6c, we did the same statistics as in Figures 4 and 5, which are shown in Figure 6d. The parameter distributions for the occurrence of phase‐2 EADs and for phase‐3 EADs and TAs show the same dependences on the maximum conductance of the ionic currents as for the other two models.

### 3.3 Roles of T‐type Ca^2+^ current

I_Ca,T_ is expressed in normal sinoatrial node cells and Purkinje fiber cells, not in normal adult ventricular myocytes (Ono & Iijima, 2010; Rosati et al., 2007; Shorofsky & January, 1992; Vassort et al., 2006). However, it has been shown that I_Ca,T_ is present in neonatal and failing ventricular myocytes (Cribbs, 2010; Ferron et al., 2003; Huang et al., 2000) as well as IPSC derived cardiac myocytes (Kernik et al., 2019). Since the voltage for I_Ca,T_ activation is much lower than that for I_Ca,L_ activation, we hypothesize that I_Ca,T_ may play a key role in the genesis of phase‐3 EADs and TAs. We incorporated an I_Ca,T_ model (Puglisi & Bers, 2001; Wang & Sobie, 2008) to the three AP models to investigate the roles of I_Ca,T_ in the genesis of phase‐3 EADs and TAs.

**FIGURE 7:**
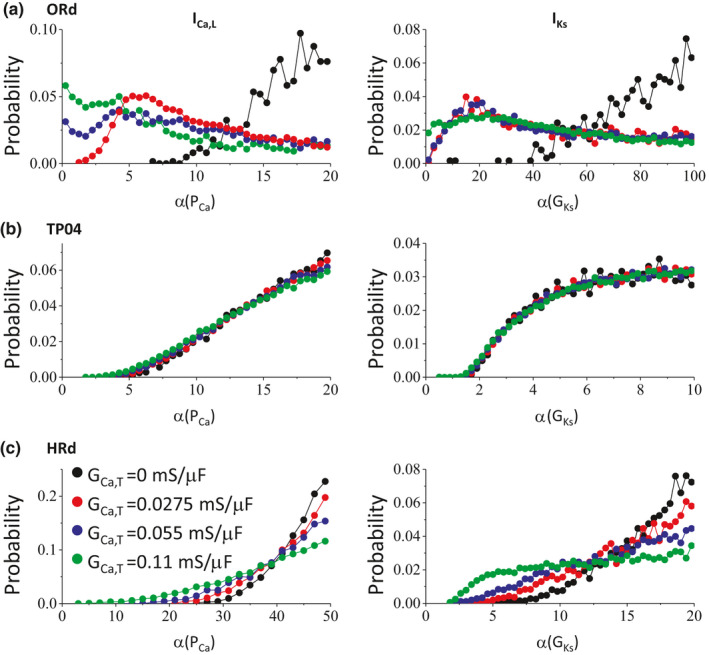
Roles of T‐type Ca^2+^ current in the genesis of phase‐3 EADs. Shown are probability of events (the fraction of the parameter sets giving rise to EADs) versus α (fold of the control value of the maximum conductance) when the I_Ca,T_ is absent (black circles) and present (colored circles). The same G_Ca,T_ values as labeled in panel c are used for all three AP models. (a) The ORd model. The total number of events (the parameter sets giving rise to phase‐3 EADs and TAs) is 617, 2,577, 5,414, and 17,347 for the four G_Ca,T_ values. (b) The TP04 model. The total number of events (the parameter sets giving rise to phase‐3 EADs and TAs) is 10,695, 35,295, 44,517, and 66,942 for the four G_Ca,T_ values. (c) The HRd model. The total number of events (the parameter sets giving rise to phase‐3 EADs and TAs) is 3,909, 3,006, 5,945, and 16,349 for the four G_Ca,T_ values

We first performed the same random parameter selections as in Figures 4, 5, 6 except that G_Ca,T_ was fixed at different values. Figure 7a shows the distributions of the maximum conductance of I_Ca,L_ and I_Ks_ for different G_Ca,T_ values for the ORd model. In the presence of I_Ca,T_, the thresholds of I_Ca,L_ and I_Ks_ for phase‐3 EADs and TAs are substantially reduced. The requirements for other currents also become less stringent. Moreover, if I_Ca,T_ is large enough, no I_Ca,L_ or I_Ks_ is needed for phase‐3 EADs and TAs. We observed similar effects in the HRd model but in a less extent (Figure 7c) and the effects in the TP04 model are very small (Figure 7b). Note that for the latter two models, the I_Ca,L_ conductance for phase‐2 EADs have to be many folds larger than the control values (see the I_Ca,L_ conductance thresholds for phase‐2 EADs in Figures 5 and 6), and it seems that adding I_Ca,T_ cannot reduce the threshold to be much lower than that for phase‐2 EADs. Moreover, in all models, phase‐3 EADs and TAs are still promoted by increasing I_Ks_ in the presence of I_Ca,T_.

**FIGURE 8:**
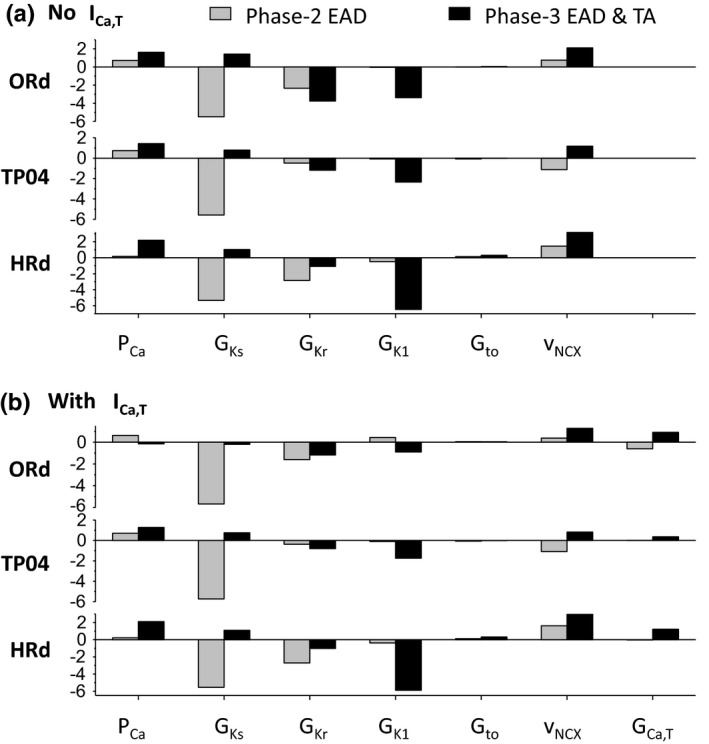
Parameter sensitivities of phase‐2 EADs and phase‐3 EADs and TAs to the maximum conductance of different ionic currents. (a) No I_Ca,T_ presence. (b) With I_Ca,T_ presence. The assigned intervals of the maximum conductance were the same as described in methods and the G_Ca,T_ range was set as [0, 0.11] mS/μF

Figure 8 shows the parameter sensitivities of phase‐2 EADs and phase‐3 EADs and TAs in the absence (Figure 8a) and presence (Figure 8b) of I_Ca,T_ using the regression methods as in previous studies (Morotti & Grandi, 2017; Sobie, 2009). In the absence of I_Ca,T_, phase‐2 EADs are positively correlated with P_Ca_ and v_NCX_, strongly negatively correlated with G_Ks_ and G_Kr_, and very weakly correlated with G_K1_ and G_to_. Phase‐3 EADs and TAs are strongly positively correlated with P_Ca_, G_Ks_, and v_NCX_, strongly negatively correlated with G_Kr_ and G_K1_. The presence of I_Ca,T_ exhibits almost no change in the parameter sensitivities for phase‐2 EADs. However, the presence of I_Ca,T_ substantially reduces the strong correlations of phase‐3 EADs and TAs with P_Ca_, G_Ks_, G_K1_, and v_NCX_ for the ORd model although it has a much smaller effects in the other two models. These correlations agree with the histograms shown in Figures 4, 5, 6, 7.

## 4 DISCUSSION

In this study, we carried out computer simulations of three ventricular AP models to investigate the cellular mechanisms of phase‐3 EADs and TAs. We show that differing from phase‐2 EADs which are promoted by increasing inward currents (e.g., I_Ca,L_) and reducing outward currents (e.g., I_Kr_ and I_Ks_), the occurrence of phase‐3 EADs and TAs requires a reduction of I_Kr_ but a substantial increase of both I_Ca,L_ and I_Ks_. Moreover, phase‐3 EADs and TAs also require a large reduction of I_K1_ and increase of I_NCX_ while phase‐2 EADs are much less sensitive to the changes of these currents. When I_Ca,T_ is present (such as in Purkinje fibers or neonatal and failing ventricular myocytes), the thresholds of I_Ca,L_ and I_Ks_ for phase‐3 EADs and TAs can be greatly reduced. Based on many previous studies on phase‐2 EADs and our present study on phase‐3 EADs and TAs, we discuss the mechanisms of the two types of EADs and the conditions for observing phase‐3 EADs and TAs in cardiac myocytes in the sections below.

### 4.1 Mechanisms of phase‐2 EADs

**FIGURE 9:**
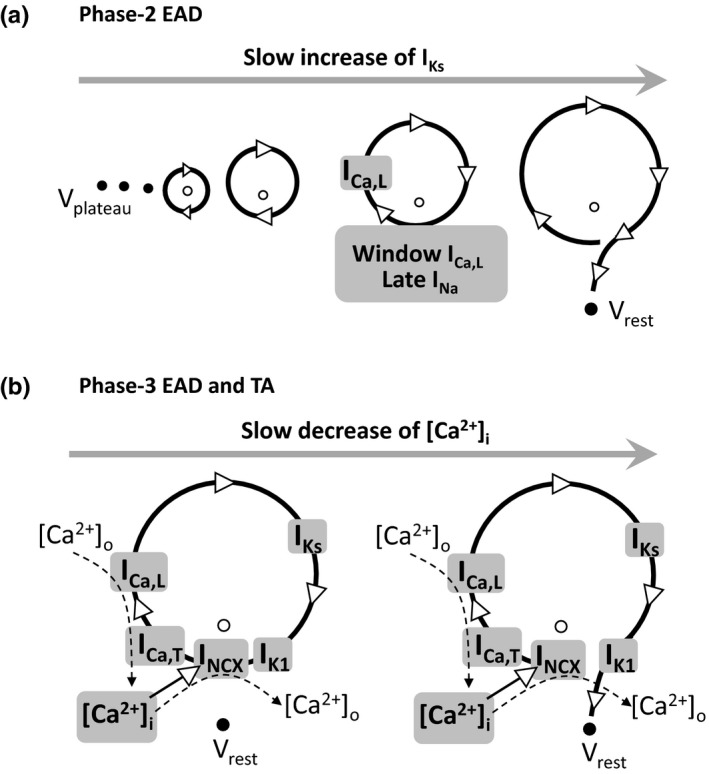
Schematic diagrams of dynamical mechanisms for phase‐2 EADs and phase‐3 EADs and TAs. See text for a detailed description. (a) Phase‐2 EADs. (b) Phase‐3 EADs and TAs. [Ca^2+^]_o_ is the extracellular Ca^2+^ concentration and [Ca^2+^]_i_ is the intracellular Ca^2+^ concentration

Phase‐2 EADs have been widely investigated in experimental and computer simulation studies and the dynamical mechanisms and the required key currents have been revealed in recent theoretical studies (Huang et al., 2018; Kügler, 2016; Kurata et al., 2017; Tran et al., 2009; Xie et al., 2014). Here we briefly summarize the mechanisms of phase‐2 EADs (with a schematic diagram in Figure 9a), which were also discussed in our previous publications (Qu et al., 2013; Song et al., 2015). The purpose is to compare the mechanism of the phase‐3 EADs and TAs with that of the phase‐2 EADs. For phase‐2 EADs, the latency of the first EAD from the AP upstroke is usually >200 ms [see table 1 in Huang et al (Huang et al., 2018)]. At 200 ms or a longer time after the AP upstroke, most of the currents reach their steady states or near their steady states except the slow ones, such as I_Ks_. The total steady‐state inward current (e.g., summation of the window I_Ca,L_, late I_Na_, and I_NCX_) and the total outward current (including the steady‐state and slowly changing outward currents) are roughly equal at the plateau voltage, forming a quasi‐equilibrium state (indicated by the filled dots in Figure 9a). As the time progresses, more I_Ks_ slowly activates, destabilizing the quasi‐equilibrium state (the open dot inside the arrowed circle) to result in oscillations. The transition from the stable equilibrium state to the unstable equilibrium state is called a Hopf bifurcation (Qu et al., 2013). The oscillation cycle can be understood as follows: the window I_Ca,L_ (or late I_Na_ and I_NCX_) prevents the voltage to decay into phase 3, and once enough LCCs recover, they open to cause voltage elevation. When voltage is high enough, the LCCs are inactivated and the outward K^+^ currents (I_Kr_ and I_Ks_) causes the voltage to decay until the window I_Ca,L_ stops further decay, forming a cycle. As I_Ks_ increases, the lowest voltage in a cycle becomes lower, causing more LCCs available to open and thus a higher peak voltage (large EAD amplitude, see I_Ca,L_ and I_Ks_ in Figure 3a). As I_Ks_ increase even further, the system approaches to another bifurcation point, called homoclinic bifurcation at which the oscillation can no longer be sustained. This is because the window I_Ca,L_ is not strong enough to compete against I_Ks_ to prevent the voltage decaying into phase 3 and thus repolarizing to the resting potential. As shown in our previous analyses (Huang et al., 2018; Qu et al., 2013; Tran et al., 2009), the key determinant of the stability of the quasi‐equilibrium steady state (the plateau phase) and the voltage oscillations (or the Hopf bifurcation) is the activation and inactivation kinetics of I_Ca,L_ with other currents playing auxiliary roles. Note that when the window I_Ca,L_ is too large, the quasi‐equilibrium state become a true equilibrium state, repolarization failure occurs. Therefore, increasing inward currents may reduce the occurrence of phase‐2 EADs, which may be the reason that increasing I_Ca,L_ conductance (e.g., Figure 6d) or I_NCX_ (e.g., Figure 5e) can also reduce the propensity of phase‐2 EADs.

Therefore, in the core of the genesis of phase‐2 EADs, the activation and inactivation kinetics of I_Ca,L_ generate the oscillations while the slow increase of I_Ks_ [can be the slow increase of I_Kr_, such as in the condition of LQT1 (Choi et al., 2018)] competes with the window I_Ca,L_ (and late I_Na_) to bring the system into and out of the oscillatory regime. Other currents play auxiliary roles in facilitating the genesis and termination of EADs.

### 4.2 Mechanisms of phase‐3 EADs and TAs

In this study, we used computer simulations to investigate the key ionic currents responsible for phase‐3 EADs and TAs, which largely agrees with the experimental conditions for phase‐3 EADs and TAs in Purkinje fibers (Bailie et al., 1988; Damiano & Rosen, 1984; Davidenko et al., 1989; Gilmour & Moise, 1996; Roden & Hoffman, 1985; Szabo et al., 1994, 1995). But surprisingly, the occurrence of phase‐3 EADs and TAs requires a substantial increase of I_Ks_, which seems to be nonintuitive based on the traditional understanding for the genesis of EADs. A rigorous analysis as for phase‐2 EADs (Tran et al., 2009) is still needed to pinpoint the mechanism and the necessary and sufficient conditions for the genesis and termination of the oscillations manifesting as phase‐3 EADs and TAs. Here we use a schematic diagram (Figure 9b) to postulate a cellular mechanism of phase‐3 EADs. The left panel in Figure 9b describes an oscillation cycle for a phase‐3 EAD or a TA. During this cycle, I_NCX_ outcompetes I_K1_ to hold the voltage at phase 3, preventing it from decaying to the resting potential. Moreover, it slowly elevates the voltage (see Figure 3b) to the threshold for I_Ca,L_ activation, at which a full depolarization occurs. This depolarization activates more outward K^+^ currents (mainly I_Ks_, see Figure 3b) which then repolarizes the cell back into the phase 3 of the AP. This cycle repeats to result in voltage oscillations manifesting as phase‐3 EADs and TAs. During this cycle, LCCs bring in Ca^2+^ from outside and NCX extrudes Ca^2+^, the two competes to maintain a proper [Ca^2+^]_i_ level to keep the cycle going. As time progresses, the amount of Ca^2+^ brought in by the LCCs remains the same in each cycle but more Ca^2+^ is extruded by NCX due to that the cycle length becomes longer and longer (see Figure 3b). This results in a decrease in the diastolic [Ca^2+^]_i_ level, and thus a smaller inward I_NCX_. Because of the reduction of I_NCX_, it can no longer outcompete I_K1_ to maintain the voltage above phase 3, and thus the oscillations terminate and the voltage decays to the resting potential (as illustrated in the right panel in Figure 9b). Sustained oscillations can be maintained when the amount of Ca^2+^ entering the cell is equal to the amount of Ca^2+^ leaving the cell, resulting in a pacemaker.

Since the activation threshold of I_Ca,L_ is high (around −40 mV), it requires an inward current to depolarize the cell from phase 3 (in the range from −70 to −60 mV) to phase 2 (above −40 mV) for I_Ca,L_ activation. This can be achieved by different ways. One way is to substantially increase the I_Ca,L_ conductance since there is still some LCCs that can be opened below −40 mV. However, a very large I_Ca,L_ conductance may result in repolarization failure, and thus a very large I_Ks_ is then needed for repolarization. This is why very large I_Ca,L_ and I_Ks_ conductances are needed for phase‐3 EADs and TAs to occur in the AP models simulated in this study. The reason that a large I_Ks_ but not I_Kr_ is required is because I_Ks_ is almost fully deactivated during phase 3 but I_Kr_ does not (see Figure 3). Therefore, if I_Kr_ is large, then the voltage will decay from phase 3 to the resting potential, suppressing phase‐3 EADs and TAs. Another way is to increase I_NCX_, but as shown in Figures 4, 5, 6, large I_NCX_ does not bring the I_Ca,L_ and I_Ks_ conductance thresholds to the normal levels. The third way is to increase I_Ca,T_. Since its activation threshold is in the range of −60 mV, it provides the inward current needed to depolarize the cell from the −60 mV range to the −40 mV range, greatly reducing the conductance thresholds of I_Ca,L_ and I_NCX_ for phase‐3 EADs and TAs. Because of the reduction of the I_Ca,L_ conductance threshold, the I_Ks_ conductance threshold is also greatly reduced. Note that if I_Ca,T_ is large enough, phase‐3 EADs and TAs can occur without the presence of I_Ca,L_ (see Figure 7).

Therefore, in the core of the genesis of phase‐3 EADs and TAs, I_NCX_ outcompetes I_K1_ to prevent the voltage from decaying from phase 3 to the resting potential. I_NCX_ and I_Ca,T_ elevate the voltage from phase 3 to phase 2 for I_Ca,L_ activation. I_Ca,L_ activation depolarizes the voltage to the peak. Activation of I_Ks_ is then required to bring the voltage back to phase 3. The process repeats to result in the oscillations manifesting as phase‐3 EADs and TAs. The slow decrease of the diastolic Ca^2+^ level due to mismatch of Ca^2+^ entry via LCCs and Ca^2+^ extrusion via NCX terminates the oscillations, repolarizing the cell to the resting potential. Differing from phase‐2 EADs in which oscillations are mainly caused by the activation and inactivation of I_Ca,L_ while I_Ks_ always remains activated, phase‐3 EADs and TAs require activation and deactivation of I_Ks_ during the oscillation cycle. Note that Figure 9b is only a schematic diagram, a rigorous mathematical analysis as for phase‐2 EADs (Huang et al., 2018; Kügler, 2016; Kurata et al., 2017; Tran et al., 2009; Xie et al., 2014) is needed to pinpoint the bifurcations leading to and the minimally ionic currents needed for the oscillations.

### 4.3 Implications for the genesis of phase‐3 EADs and TAs in ventricular myocytes

Phase‐3 EADs and TAs have been widely observed in isolated Purkinje fibers (Damiano & Rosen, 1984; Davidenko et al., 1989; Gilmour & Moise, 1996; Roden & Hoffman, 1985; Szabo et al., 1994, 1995). These experiments have shown that phase‐3 EADs and TAs are promoted by hypokalemia and blocking outward currents with Cs^+^ (Bailie et al., 1988; Damiano & Rosen, 1984; Szabo et al., 1994, 1995) or quinidine (Davidenko et al., 1989; Roden & Hoffman, 1985). However, the type of phase‐3 EADs and TAs as shown in Figure 1b has been rarely reported in experiments of isolated ventricular myocytes. Phase‐3 EADs and TAs were observed in ventricular tissue experiments (Bailie et al., 1988; Ben‐David & Zipes, 1988; El‐Sherif et al., 1990; Hwang et al., 2020), however, it is unclear if they were oscillations caused by a single‐cell mechanism or by a tissue‐scale mechanism. As shown in both simulation and experimental studies (Huang et al., 2016; Maruyama et al., 2011), phase‐3 EADs and TAs can be generated by a tissue‐scale instability due to heterogeneous repolarization. Late phase‐3 EADs and TAs have been shown in atrial and ventricular tissue (Burashnikov & Antzelevitch, 2003; Maruyama et al., 2014), which are caused by Ca^2+^ overload and lengthening of the Ca^2+^ transient duration. This mechanism can only give rise to a single TA after a regular AP, not multiple TAs or oscillations as shown in Figure 1b. Another type of phase‐3 EADs has been observed in mouse ventricular myocytes (Edwards et al., 2014) and rat atrial and ventricular myocytes (Tazmini et al., 2020). Nonequilibrium reactivation of I_Na_ was shown to drive this type of EADs (Edwards et al., 2014; Morotti et al., 2016), which was observed for triangulated APs with short APDs, such as for mice and rats. However, no phase‐3 EAD induced TAs (as the ones in Figure 1b) were observed, and the features of this type of phase‐3 EADs, such as the EAD amplitude and oscillation period, are similar to those of phase‐2 EADs despite lower takeoff potentials (from −55 to −35 mV). Another type of phase‐3 EADs was shown in computer simulations (de Lange et al., 2012; Xie et al., 2010), which was generated by adding a Ca^2+^‐activated nonselective cation current [I_ns(Ca)_] to the AP model. Although I_ns(Ca)_ has been identified in different species (Doerr et al., 1990; Ehara et al., 1988; Giles & Shimoni, 1989), its existence is controversial and its physiological or pathophysiological roles remain unclear.

Our current simulations demonstrate that in the absence of I_Ca,T_, besides lower I_K1_ and I_Kr_ conductance, it requires a very large I_Ca,L_ conductance and a very large I_Ks_ conductance for phase‐3 EADs and TAs. But in the presence of I_Ca,T_, the thresholds of these two currents can be greatly reduced. However, in normal ventricular myocytes in which no I_Ca,T_ is present, based on our simulations, it will require a very large I_Ca,L_ conductance and a very large I_Ks_ conductance to cause phase‐3 EADs and TAs. These conductances may be too large to be realistic in real ventricular myocytes. This may explain why no phase‐3 EADs and TAs have been observed in isolated ventricular myocyte experiments. On the other hand, I_Ca,T_ is expressed in neonatal and failing ventricular myocytes. Under hypokalemia (which lowers I_K1_) and infusion of isoproterenol [which increases both I_Ca,L_ and I_Ks_ (Liu et al., 2012), and loads the cell with more Ca^2+^ to increase I_NCX_], phase‐3 EADs and TAs may be observed in neonatal and failing ventricular myocytes. In fact, phase‐3 EADs and TAs were observed in IPSC derived cardiac myocytes with infusion of isoproterenol (Spencer et al., 2014), which may benefit from the presence of I_Ca,T_ (Kernik et al., 2019). Since I_Ca,T_ is expressed in Purkinje fiber cells (Ono & Iijima, 2010; Rosati et al., 2007; Shorofsky & January, 1992; Vassort et al., 2006), it may also play an important role in phase‐3 EADs and TAs in Purkinje fiber cells. However, the ionic currents differ substantially between Purkinje fiber cells and ventricular myocytes, whether I_Ca,T_ is key to the genesis of phase‐3 EADs and TAs in Purkinje fiber cells needs to be investigated in future studies.

## 5 LIMITATIONS

Although we obtained the same general conclusions from the simulations of three widely used AP models, they do exhibit differences. For example, their susceptibilities to phase‐2 EADs and to phase‐3 EADs and TAs are very different, and their responses to I_Ca,T_ are also very different. Future studies are needed to pinpoint the causes giving rise to these differences, which will be helpful for a better understanding of the mechanisms of phase‐3 EADs and TAs in ventricular myocytes. We only simulated three AP models, our conclusions need to be examined in other AP models, and in particular validated in future experiments.

## 6 CONCLUSIONS

Our computer simulations of ventricular myocyte models show that besides the known promoting factors (e.g., blocking I_K1_ and increasing I_NCX_), extensive increase of both I_Ca,L_ and I_Ks_ is needed for the genesis of phase‐3 EADs and TAs in the absence of I_Ca,T_. However, in the presence of I_Ca,T_, these requirements can be substantially weakened. Our results imply that it will become easier to observe phase‐3 EADs and TAs in neonatal and failing myocytes as well as IPSC derived cardiac myocytes where I_Ca,T_ is present. Due to the importance of I_Ca,T_ for the genesis of phase‐3 EADs and TAs, I_Ca,T_ may play an important role in arrhythmogenesis in cardiac diseases.

## AUTHOR CONTRIBUTIONS

ZQ conceived the overall study and drafted the manuscript, ZZ performed the simulations, ZQ and ZZ analyzed the data and edited the manuscript.

## Data Availability

The data and source codes that support the findings of this study are available from the corresponding author upon reasonable request.
